# A Doctor "On the Stump"

**Published:** 1890-05-10

**Authors:** 


### The Doctor's Pulpit.

### A DOCTOR "ON THE STUMP."

"In arguing, too, the parson own'd his skill,
For e'en though vanquish'd, he could argue still;
While words of learned length and thund'ring sound
Amazed the gazing rustics ranged around;
And still they gazed, and still the wonder grew
That one small head could carry all he knew."

Happy pedagogue! Thy epitaph is written by the sweetest of poets, and thou hast no successor of thine own order! The mantle of the village schoolmaster has fallen upon the village doctor. The portentous literary pomp of Samuel Johnson having gone the round of the Established and Nonconforming pulpits, of the village school and the provincial newspaper office, seems to have found a final resting-place, in a miserably degraded form, in the surgery of the medical practitioner. On more than one occasion it has come within the range of our duty to describe a medical lecturer in these pages. We have seldom been able to speak with unqualified commendation either of the simplicity of his style or of the suitability of his subject-matter. But a lecture by a medical man which recently appeared in one of the Dorsetshire newspapers surpasses all previous attempts in the pompous turgidity of its diction and the futile inconsequence of its logic. It would not really be worth while to deal with this lecture if it stood alone. But doctors seem to have been recently smitten with a lecturing mania; and it becomes of some importance to medical men as a class that those of their number who take to "stump oratory" should not bring discredit on the whole profession.

By way of justification of this preface a few samples of our lecturer's medico-scientific eloquence will here be given. Many persons speak slightingly of Macaulay nowadays; but the present writer would be willing to pay a very handsome fee for the use of his pen for two or three hours. The lecturer, whom we will not more particularly name, discoursed to the amazed rustics of his Dorsetshire village on "The physiological conservation of energy and its expenditure in the production of work." "Proud title," as Verdant Green said when under the inspiring influence of his fourteenth glass of college port: "Proud title." Wondrous title indeed for a village lecture. People talk of the power of the printed page; but what must the carpenter, the tailor, hob-nailed Hodge, and even the schoolmaster have thought of Dr. —— when he sent round his circular inviting them to a literary feast at which the *pièce de résistance* was to be "The physiological conservation of energy and its expenditure in the production of work"? Would it not have been possible to have named the lecture in some such terms as the following: "Nature's thrift: and her way of spending"? The people of the village would then at least have had an idea put into their heads. But we feel certain that so far as nine-tenths of them were concerned Dr. —— might just as well have called his lecture τυπτω, τυπτεις, τυπτει, or πασ, πασα, παν.

Here is another flower of medico-scientific rhetoric. Said the Doctor, "Work might be roughly defined as a process effecting a change of place, or a change in the condition of matter, bringing about phenomena the natural outcome of such changes. Energy, in its turn, might be defined as an imponderable element of nature, associated with all kinds of matter, but, as being extraneous to it, not a part of it, matter representing the medium by which energy could be stored and conducted." No doubt! And a pig may be defined as a "curved-backed proboscidean, with a twisted caudal appendage, four lower extremities having ungulate terminations, an omnivorous digestive apparatus, and a tendency to develop into bacon and ham in an appropriate environment." It may also for country folk be defined as a "grunter"; and it is quite probable that the rustic will understand the latter definition at least as well as the former. Does the doctor really believe that the excellence of a popular lecture consists in making easy things difficult and simple things obscure? Or does he suppose that even half-a-dozen of his audience were tricked into believing him a learned and scientific man by the superabundance of his ponderous and pompous phraseology? If the parson did him the honour of being present we may be sure that he, at least, had a pin ready to thrust into the wordy wind-bag; and if a single intelligent schoolboy were there, he, too, would know that the lecturer was attempting to fly without having first provided himself with wings, and that the only possible result would be a discreditable and ignominious fall.

The whole lecture, which must have occupied not less than an hour in delivery, is of a piece with the specimens already given. One more extract may suffice for the present. Said the lecturer: "Thus they might say, with literal truth, that every kind of work performed by man, whether by the use of other animals, or by that of machinery, or by the use of his own energy, of either mind or body, was executed by the force of latent sunshine conservated or 'moleculed' to his hand by the potent protoplasm of plant life." This, if you please, is the doctor's way of teaching science; this the method of expounding the new learning which is to take the place, as some fondly hope, of history and of ancient and modern literature in the instruction and development of the minds of the future; this miserable farrago of the smallest of ideas expressed in the biggest of words, and the most sesquipedalian of sentences. It is thus that the modern medical man delights to pose and posture before his patients and the world. Small wonder is it if, here and there, a keen and sarcastic intellect declares that not only has medicine always been in the past, but that it still is, a mass of ignorant pretence and vulgar charlatanism.

The modern doctor we admit, nay, we contend, has much to teach, and much that, what scientists patronisingly call "the man in the street," eagerly desires to learn. But teaching is a skilled art, an art in which no headway whatever can be made by a masquerading harlequin whose only desire is to show his audience the stripes on his literary trousers and the red and white chalk on his scientific cheeks. If country doctors wish for models on which to frame their lectures they cannot do better than take the sermons of such men as Dr. Vaughan, of the Temple Church, or of many a country clergyman whose learning often as much exceeds that of the ordinary medical man as the medical man's is ahead of the brightest village schoolboy's. Those men, of high and genuine culture, prove the reality and value of that culture by illuminating as with the rays of the sun every subject they expound; by making rough places smooth, obscure things plain, and difficult things easy. To them big words, and long, involved, and sounding sentences are as hateful as the loud manners of the bookmaker or the checked trousers and flaming tie of a cockney 'Arry out for a 'oliday. Every medical man who speaks in public should remember that he speaks not for himself only, but also for the whole medical profession. The doctor who regards his profession with the honour and esteem with which he ought to regard it will take care that so far as he is concerned that profession shall always appear to the public clothed with simplicity, with true, if not with deep learning, and with the severest scientific honesty. In this way not only will medicine win honour; but real and valuable knowledge will enlighten the general mind and, promote both well-being and happiness.

